# P08-17 Physical activity according to migration status in adolescents living in French-speaking Belgium

**DOI:** 10.1093/eurpub/ckac095.130

**Published:** 2022-08-29

**Authors:** Emma Holmberg, Camille Pedroni, Thérésa Lebacq, Véronique Desnouck, Maud Dujeu, Katia Castetbon

**Affiliations:** 1 Service d'Information, Promotion, Education Santé (SIPES), Research Centre in ‘Epidemiology, Biostatistics and Clinical Research', School of Public Health, Université Libre de Bruxelles, Brussels, Belgium; 2 Research Centre in ‘Social Approaches to Health', School of Public Health, Université Libre de Bruxelles, Brussels, Belgium

**Keywords:** Physical activity, Adolescents, Immigration status, Youth, Sociodemographic disparities

## Abstract

**Background:**

Rising levels of childhood obesity is a worldwide concern, with physical inactivity considered to be amongst the many contributors. Worryingly, physical activity (PA) tends to decline throughout adolescence. Although there is extensive research on the sociodemographic disparities of adolescent PA participation, less evidence is available on the potential involvement of immigration status in such disparities. The aim of this study was to investigate PA levels according to migration status among adolescents aged 12-20 years in Belgium.

**Methods:**

This study used the data from the cross-sectional 2018 Health Behaviour in School-aged Children (HBSC) survey in French-speaking Belgian schools (Brussels and Wallonia). A two-stage random sample was used to select participants. Data was collected using self-administrated questionnaires. Adolescents aged 12 to 20 were included in the analyses presented here (n = 8635, boys: n = 4179, girls: n = 4456). The association of global PA (GPA) and vigorous PA (VPA) with migration status (natives, 2nd and 1st-generation immigrants) was analysed using multiple binary logistic regression analyses. Interactions with gender were tested.

**Results:**

The prevalence of adolescents undertaking sufficient GPA (moderate to vigorous PA 60 minutes/day and VPA ≥3 times/week) was higher amongst 1st-generation immigrants (11.7%) compared to 2nd-generation immigrants (7.4%) and natives (8.9%) (p = 0.01). Vigorous PA ≥ 3 times/week was significantly more prevalent amongst natives (52.6%) than 2nd (44.9%) and 1st (48.8%) generation immigrants. After adjusting for sociodemographic variables, compared to natives, 2nd-generation immigrants were less likely to participate in sufficient GPA (aOR= 0.83, 95%CI: 0.69-1.00) and VPA (aOR= 0.77, 95%CI: 0.68-0.87). Conversely, 1st-generation immigrants were more likely to be sufficiently active compared to natives (GPA: aOR= 1.44, 95%CI: 1.03-2.01). An interaction between migration status and gender was found for VPA only (p > 0.001). Compared to natives, 1st-generation immigrant boys were more likely (aOR=1.42, 95%CI: 1.15-1.75) and immigrant girls were less likely to undertake VPA ≥3 times/week (2nd-generation: aOR= 0.66, 95%CI: 0.56-0.78; 1st-generation: aOR=0.72, 95%CI: 0.57-0.90).

**Conclusions:**

This study shows disparities in PA participation according to migration status and gender, independently of sociodemographic characteristics, for adolescents living in French-speaking Belgium. These findings will enable to inform future public health initiatives promoting PA in adolescents on migration and gender-specific considerations.

